# Increasing access to psychological treatments for adults by improving uptake and equity: rationale and lessons from the UK

**DOI:** 10.1186/s13033-018-0246-7

**Published:** 2018-11-09

**Authors:** June S. L. Brown

**Affiliations:** Psychology Department, King’s College London, Institute of Psychiatry, Psychology and Neuroscience, De Crespigny Park, London, SE5 8AF UK

**Keywords:** Access, Equity, Uptake, Self-referral, “Group-sensitive engagement”, Unmet need

## Abstract

**Objectives:**

Access to psychological treatments has been defined by Gulliford as comprising supply, effectiveness, equity and uptake. In the UK, a recent national programme “Improving Access to Psychological Treatments” has significantly increased supply and assessed effectiveness, but paid less attention to uptake and equity. The model developed by Gask et al. delineating processes relevant to improving access for ‘hard-to-engage’ groups in the UK, including black and minority groups seems relevant. This paper presents studies of a large-scale ‘community workshop’ intervention model developed by Brown to improve access for adults in the UK, designed to improve uptake and equity. We describe two ‘community workshop’ interventions for common mental health problems to which people have been able to self-refer and where uptake and equity have been high. Key components of this model are a ‘group-sensitive engagement’ ethos which includes self-referral, non-diagnostic titles of the intervention, a non-mental health setting, face-to-face presentation as well as a brief intervention and an acceptable format.

**Conclusion:**

The model of community workshops with its ‘group-sensitive engagement’ ethos to which adults can self-refer may be very relevant in providing access for people with mental health needs in national and international settings.

*Trial registration* Classic ISRCTN26634837

## Introduction

Since 2009 the UK Government has invested £400 million in psychological services so that the IAPT programme has dramatically increased the resources available to people with mental health problems. The rationale was that the provision of more evidence-based psychological treatments could ultimately save public money, through reductions in unemployment and social welfare costs normally associated with untreated (or undertreated) mental health problems. Key targets have been an increased workforce trained to deliver cognitive-behavioural therapy (CBT) and other evidence-based treatments for common mental health problems as recommended in national clinical guidelines and good clinical outcomes [1].

Overall, more than 1 million people were seen in IAPT services within the first 3 years of the IAPT programme, with more than 680,000 completing a full course of psychological therapy. Recovery rates were in excess of 45% with nearly 250,000 cases (41%) recovering, and around two-thirds showing reliable improvement [1]. IAPT now offers treatment to more than 900,000 service users each year, with a target for at least 1.5 million people to access psychological care each year by 2020/21 [2].

However, although generally effective in head-to-head comparisons in research studies, not all psychological treatment formats are equally popular on the uptake side in services. Options in IAPT include face-to-face therapy, guided self-help using workbooks, reading recommended books and computerised delivery. The latter may involve either a ‘blended’ approach with face-to-face sessions taking place alongside online content delivery, or else may be restricted entirely to digital methods. While computerised CBT may appear to be both an effective and convenient option for some people, uptake appears low and dropout relatively high, with only a median of 56% completing the full course [3]. Because of the low uptake, this has led some IAPT services stopping providing computerised CBT.

In terms of equity, quarterly figures indicate that the proportion of IAPT users from minority ethnic groups is lower than expected [4]. In addition, the completion rates (13.4% versus 49.7%) and recovery rates (40.2% versus 45.4%) of these groups appear lower than for other ethnic groups [4].

## Some theoretical work around access to health care

Gulliford et al. [5] identified four main dimensions of access to health care: (a) adequacy of supply and services (b) utilisation of services—or uptake (c) effectiveness and relevance of services and (d) equity of services to meet the needs of different groups. It can be argued that although IAPT has been extremely successful in increasing the first (supply) and demonstrating the third (effectiveness) aspects, two other key aspects, uptake and equity are not addressed so well.

Gask et al. [6] have developed a process model about access to mental health care for “difficult to engage” groups. Very broadly, a multifaceted model to improve access to mental health care is proposed:

- Stage 1: *Community engagement:* Engaging local community stakeholders to better understand the attitudes and beliefs of community members to develop more responsive and sustainable services.
- Stage 2: *Primary care quality:* Helping primary care staff to help patients feel ‘listened to’, to gain more of a ‘shared narrative’ through training in cultural competence and patient explanatory models.
- Stage 3: *Psychosocial interventions*: Designing interventions that are tailored to the preferences of underserved groups to increase acceptability, whilst ensuring that core evidence-based mechanisms are not lost.

### Community workshops

Drawing on the above, Brown has developed a model of large-scale “community workshops” [7], (each for up to 30 adults) designed to reduce barriers to equity and uptake of mental health care. As only about 30% of adults with mental health problems use services, Brown has utilised a ‘group-sensitive engagement’ ethos to increase uptake and equity and argued that, as well as being effective, accessible interventions should fit with people’s preference for easy access to brief, practical help at convenient times and in non-mental health locations. Two RCTs that have evaluated community workshops using this model will be described, followed by a summary of the key ingredients relevant to increasing uptake and improving equity.

## Community CBT stress workshops

As part of a world-wide WHO campaign to improve the health of the general population in cities in 1996, Brown et al. first used a self-referral route to offer access to day-long community Stress “workshops” run at the weekend in leisure centres. The workshops were very popular attracting 176 participants after 3 months’ publicity and 101 were followed up at 3-month follow-up. The workshops showed reasonably good efficacy for decreasing stress for the general population sample in an RCT (effect size 0.3). They found that 39% had not consulted their GPs suggesting that the self-referral route to an intervention could significantly increase uptake of services by people who were previously reluctant to seek help. Unfortunately, ethnicity data was not collected.

### Community CBT workshops for depression

Brown et al. then offered “depression” workshops using a similar self-referral model but found take-up was very low (n = 45), and that most of the self-referrers (90%) had already used services.

They therefore decided to ‘re-label’ these workshops as ‘self-confidence’ workshops given the strong relationship between depression and self-esteem. The rationale was that a lay label was preferable to a psychiatric ‘diagnosis’, as members of the public would understand what was being offered. The result was that many more people (n = 134) referred themselves in a 2 month period with 120 randomised into the trial. The effectiveness of the workshops with the general population sample was reasonably good (effect size 0.31) with significantly lower depression and distress at 3-month follow-up. In addition, a 2-year follow up showed a preventative effect, particularly for those with depressive level symptoms. Equity was good with 29% from BME groups, which was reasonably representative of the local community. Of the self-referrers, 39% were non-consulters again indicating that this approach increases uptake by those who had been reluctant to seek help. In a separate study, Brown et al. found that 75% of those self-referring had diagnosable problems, indicating reasonable accuracy in self-identification of problems.

An RCT of these popular self-confidence workshops but restricted to people with depression (n = 459) found significantly lower depression scores among the sample of 459 participants (effect size 0.55) at 3 month follow-up [8]. Notably, 25% of participants were GP non-consulters, indicating that those with severe problems were willing to self-refer. The intervention was also promising when cost-effectiveness was calculated. A very high uptake (32%) from different minority ethnic groups was found, this being approximately 1.5 times greater than the proportion of black and minority ethnic residents in the local population in some boroughs. Notably, outcome did not differ between ethnic groups (African, Asians, white British) even though no specific cultural adaptations were made.

## Key components of community workshops

### Community (or Group-Sensitive) engagement (Stage 1)

1. *Self-referral*

Self-referral essentially encourages individuals to decide to enrol for a particular intervention that is publicized, without having to go through a health professional. Given problems of stigma and the low level of knowledge about treatment effectiveness, the publicity needs to be eye-catching, non-stigmatising and informative about the benefits of attending the workshop.

Referral methods within IAPT have been found to significantly affect equity [9]. A comparison of GP and self-referrals to IAPT in one London borough showed that self-referral led to greater equity on ethnicity as well as age, gender and social welfare status, when compared to the local population [9].

With respect to Gask et al. access model [5], self-referral essentially focuses on community engagement (Stage 1) and to some extent on Psychosocial Intervention (Stage 3) but misses out the Primary Care Quality stage (Stage 2). Interestingly, Gask et al. [6] suggest that this combination could be more effective in attracting different minority ethnic groups, who often are reluctant to consult their GPs.

2. *Non-diagnostic titles of interventions*

These are very important, given our experience with the Self-confidence workshops. The social marketing of interventions needs to relate to ‘normal’ problems that people understand rather than diagnostic terms. Information needs to be relevant to what people are experiencing and also give ‘hope’ [8]. The titles used in the workshops have been ‘Stress’ and ‘Self-confidence’ for adults. Non-diagnostic labels and explanatory models that emphasise social rather than biomedical constructions are important in reducing anticipated stigma from potential participants.

3. *Non-mental health locations*

Locations need to be carefully chosen. Libraries and leisure centres have been used as these may be viewed as more ‘normal’ and less stigmatising.

4. *Face to face presentation*

There is evidence that face-to-face interventions are preferred to computerised interventions because they are more credible, are motivational, and offer personal support [10].

### Psychosocial intervention (Stage 3)

5. *Brief CBT group intervention*

Evidence-based CBT principles have been used in the programmes—and which have been evaluated to ensure effectiveness of the interventions. Participants are taught coping skills and techniques which are demonstrated in a very practical way during the workshop using a “psycho-educational” format. Participants are encouraged to identify personal goals and use the methods taught. While community interventions may sometimes be delivered by non-professional leaders, these community CBT workshops have been delivered by clinical psychologists with help from assistant psychologists.

6. *Acceptable format*

The interventions have been designed to be acceptable and convenient; day-long sessions at the weekend are often easier to attend than weekly sessions during the week. The programmes have been designed to be very interactive and the language used is normalised.

## Conclusion

The above model of Brown could inform how accessible services for different groups to increase uptake and improve equity can be effectively delivered in the UK as well as other countries globally, and where decisions need to be made about how members of the public access services.
